# [Retracted] miR-367 enhances the proliferation and invasion of cutaneous malignant melanoma by regulating phosphatase and tensin homolog expression

**DOI:** 10.3892/mmr.2026.13809

**Published:** 2026-01-26

**Authors:** Jianwen Long, Jing Luo, Xuwen Yin

Mol Med Rep 17: 6526–6532, 2018; DOI: 10.3892/mmr.2018.8663

Following the publication of the above paper, it was drawn to the Editor's attention by a concerned reader that the cell invasion and migration assay data shown in Fig. 5B were strikingly similar to data appearing in different form in another article written by different authors at different research institutes that had already been published in the journal *PLoS One*, albeit the brightness and contrast of the images appeared to have been altered.

Owing to the fact that the contentious data mentioned above had already apparently been published previously, the Editor of *Molecular Medicine Reports* has decided that this paper should be retracted from the Journal. The authors were asked for an explanation to account for these concerns, but the Editorial Office did not receive a reply. The Editor apologizes to the readership for any inconvenience caused.

